# Marine Collagen from European Sea Bass (*Dicentrarchus labrax*) Waste for the Development of Chitosan/Collagen Scaffolds in Skin Tissue Engineering

**DOI:** 10.3390/md23100375

**Published:** 2025-09-25

**Authors:** Alessandro Coppola, Maria Oliviero, Noemi De Cesare, Nello Russo, Noemi Nappo, Carmine Buonocore, Gerardo Della Sala, Pietro Tedesco, Fortunato Palma Esposito, Christian Galasso, Donatella de Pascale, Ugo D’Amora, Daniela Coppola

**Affiliations:** 1 Department of Ecosustainable Marine Biotechnology, Stazione Zoologica Anton Dohrn, Via Ammiraglio Ferdinando Acton 55, 80133 Naples, Italy; alessandro.coppola@szn.it (A.C.); noemi.nappo@szn.it (N.N.); carmine.buonocore@szn.it (C.B.); gerardo.dellasala@szn.it (G.D.S.); pietro.tedesco@szn.it (P.T.); fortunato.palmaesposito@szn.it (F.P.E.); donatella.depascale@szn.it (D.d.P.); 2 Department of Chemical, Biological, Pharmaceutical and Environmental Sciences, University of Messina, Viale Ferdinando Stagno D’Alcontres 31, 98166 Messina, Italy; 3 Institute of Polymers, Composites and Biomaterials, National Research Council, CNR-IPCB, 80055 Portici, Italy; maria.oliviero@cnr.it (M.O.); nellorusso@cnr.it (N.R.); 4 Institute of Polymers, Composites and Biomaterials, National Research Council, CNR-IPCB, 80125 Naples, Italy; noemidecesare@cnr.it; 5 Department of Ecosustainable Marine Biotechnology, Calabria Marine Centre, CRIMAC (Centro Ricerche ed Infrastrutture Marine Avanzate in Calabria), Stazione Zoologica Anton Dohrn, C. da Torre Spaccata, 87071 Amendolara, Italy; christian.galasso@szn.it

**Keywords:** marine collagen, chitosan, scaffold, tissue engineering, fishing discards, circular bioeconomy

## Abstract

Over the past years, with the growing interest in sustainable biomaterials, marine collagen has been emerging as an interesting alternative to bovine collagen. It is more easily absorbed by the body and has higher bioavailability. In this study, collagen was extracted from *Dicentrarchus labrax* (sea bass) skin, a fishery by-product, thus valorizing waste streams while reducing environmental impact. To overcome the intrinsic weak mechanical of collagen, it was combined with chitosan to produce composite scaffolds for skin tissue engineering. The incorporation of collagen proved crucial for scaffold performance: (i) it promoted the formation of an open-pore architecture, favorable for cell infiltration and proliferation; (ii) it enhanced swelling behavior suitable for exudate absorption and maintenance of a moist wound environment; (iii) by tuning the chitosan/collagen ratio, it enabled us to control the degradation rate; (iv) it conferred antioxidant properties; and (iv) by adjusting collagen/chitosan concentrations, it allowed fine-tuning of mechanical properties, ensuring sufficient strength to resist stresses encountered during wound healing. In vitro assays demonstrated that the scaffolds were non-cytotoxic and effectively supported mouse adipose tissue fibroblasts’ adhesion and proliferation. Finally, all formulations exhibited marked bactericidal activity against the human pathogen *Staphylococcus aureus* and the methicillin-resistant *Staphylococcus aureus,* with a Log reduction greater than 3 (a reduction of at least 99.9% in bacterial growth) compared to the control. Collectively, these findings highlight collagen not only as a sustainable resource but also as a functional component that drives the structural, physicochemical, biological, and antimicrobial performance of chitosan/collagen scaffolds for skin tissue engineering.

## 1. Introduction

The skin represents the largest organ of the human body and is the first barrier that protects the individual from external insults. Following damage, a skin lesion is autonomously repaired through biological actions that lead to complete healing [1]. However, in case of damage such as severe burns, where the tissue is unable to regenerate on its own [2,3], tissue engineering represents a promising solution [4].

Scaffold design plays a pivotal role in advancing skin tissue engineering. Scaffolds, typically made of polymeric biomaterials, provide structural support for cell attachment and subsequent tissue development. Among the biomaterials, collagen has been widely used in the realm of skin tissue engineering [5,6,7]. It is the most abundant protein in nature [8] and is a major component of the extracellular matrix (ECM) in organisms [9]. It is an extremely versatile protein thanks to its biocompatibility and biodegradability, and it has been extensively studied in the cosmetics industry for its anti-ageing properties. Indeed, collagen-based creams have been widely used to improve skin texture by counteracting the signs of ageing [10]. Collagen, characterized by a triple-helical peptide chain structure, is able to control several cell–material interactions that influence intracellular signalling and eventually cell functions such as morphogenesis, ECM deposition, and tissue remodelling [6]. However, collagen has many drawbacks despite its benefits as a biomaterial for scaffold manufacture. It is often derived from animals, such as pigs or cows, which raises questions about bacterial contamination, allergic responses, and the spread of diseases, such as bovine spongiform encephalopathy [5]. Furthermore, some patients could not accept collagen skin substitutes made from animals due to societal and religious considerations. In this context, marine-origin collagen represents an additional choice due to its exceptional biocompatibility, low antigenicity, and high degradability [11,12]. Moreover, it is universally acceptable across all religions and has not been associated with any known transmissible diseases.

Recently, fishery by-products, such as skin, scales, bones, fins, and heads, are gaining more attention as rich and yet underutilized sources of high-value bioactive compounds like collagen. Their applications fit perfectly with the principles of the circular bioeconomy and zero-waste policies, which seek to reduce waste by reusing and valorizing resources [11,13], contributing to the development of new solutions with a reduced environmental impact [14,15,16].

Despite growing interest in marine collagen extracted from fishing discards, few studies have been conducted so far on the production of fish collagen-based scaffolds for medical applications. Interestingly, fish collagen has shown interesting results in tissue engineering applications, including skin, bone, and cartilage regeneration [17,18,19,20,21]. Wound regeneration also plays a crucial role in biomedical research [22,23], where scaffolds based on fish collagen are very promising [24,25,26,27].

For this reason, in this study, we extracted the European sea bass (*Dicentrarchus labrax* (Linnaeus 1758) skin collagen (COL), as an underlooked source of marine collagen for tissue engineering. *D. labrax* is an economically important species widely present in the Mediterranean Sea, farmed and fished globally [28,29], which therefore produces a large amount of waste from fish processing and aquaculture companies, as well as from the fish market.

Nonetheless, marine collagen still shows some drawbacks, such as poor mechanical strength, aminoacidic composition, and biodegradation [5]. It lacks sufficient structural robustness when employed in three-dimensional (3D) structures, and consequently, it degrades quickly, losing stability over time. Blending with other biomaterials, such as chitosan (CS), and the use of crosslinkers can enhance lifetime and structural properties [6].

Chitosan is derived from the deacetylation of chitin, which occurs in crustaceans’ chitinous exoskeleton (i.e., shrimp, crab, and lobster) and is the most abundant cationic polysaccharide in the world [30,31]. Thanks to its structure, CS is extremely useful for providing stability and compactness to collagen, which alone would be inefficient from a structural point of view [32]. Due to their exceptional biostimulative characteristics, as well as good biocompatibility and cytocompatibility, biodegradability, non-toxicity, low immunogenicity, and also the antibacterial and immunomodulatory properties [31,33], CS and its derivatives have been used as attractive polysaccharide candidates due to the formation of complexes with other macromolecules [6,34]. Furthermore, CS is similar to the glycosaminoglycans found in the ECM. It can provide structural support for cell adhesion, migration, and growth in addition to serving as a vehicle for controlled drug release to help tissue survival and regeneration. Indeed, CS has pKa of around 6.3 and 6.5. For this reason, at lower pHs, dissolution phenomena may allow the release of growth factors and/or drugs to stimulate the last phases of the wound healing process. Meanwhile, at higher pHs, it is stable treating bacterial infection and inflammation in the first stage. However, tackling bacterial therapy and favoring tissue remodeling still represents a big challenge in scaffold design for wound healing applications.

**Figure 1:**
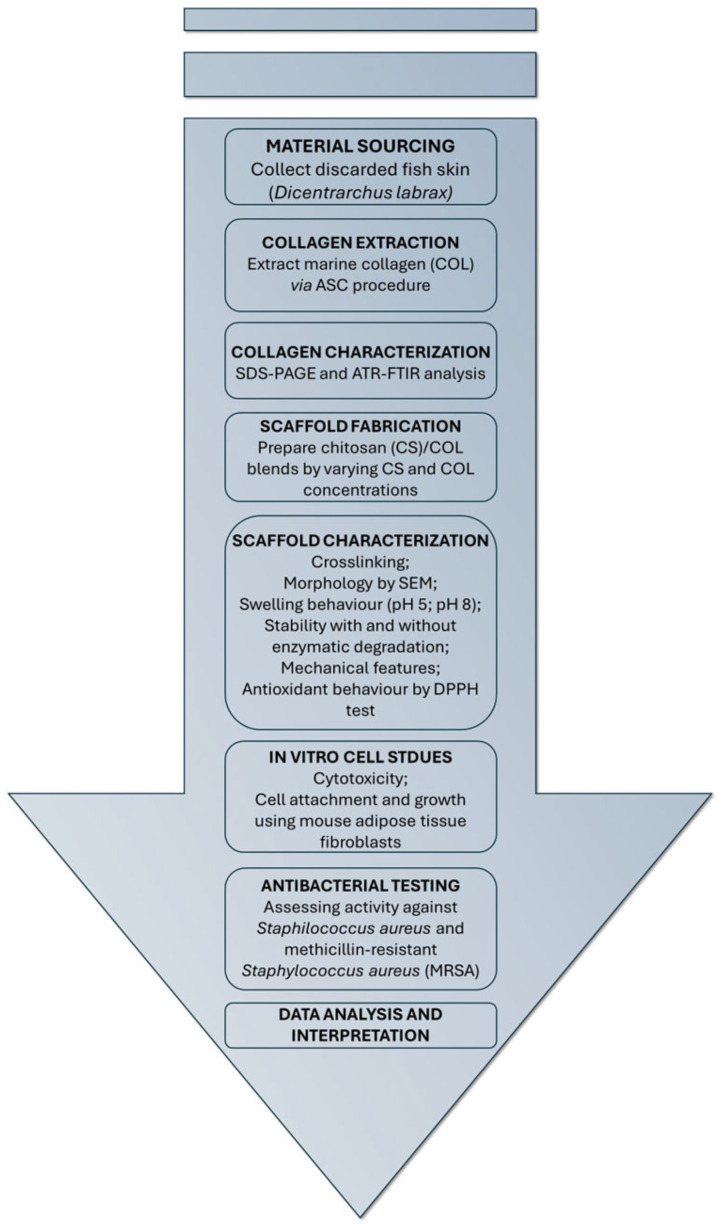
Workflow diagram outlining the study design from the material sourcing phase to the chitosan (CS)/collagen (COL) scaffold design and characterization steps.

Herein, this study aimed to evaluate the effect of the addition of CS on the morphological, physicochemical, antioxidant and mechanical properties of marine collagen-based scaffolds, to determine if COL extracted from *D. labrax* might serve as a viable alternative to bovine collagen, in skin tissue engineering application. Furthermore, swelling at pathological and physiological pH, as well as stability tests, with or without enzymatic degradation, were also investigated. A biological characterization was carried out to study toxicity and cell proliferation by using mouse adipose tissue fibroblasts (L929 cells) as cell model. Finally, antimicrobial properties against the human pathogens *Staphylococcus aureus* ATCC 6538, and the methicillin-resistant *Staphylococcus aureus* (MRSA), were also assessed to prove their potential use in skin wound healing. A workflow diagram outlining the study design is reported in Figure 1.

## 2. Results and Discussion

### 2.1. Collagen Extraction from Sea Bass Skin

**Figure 2:**
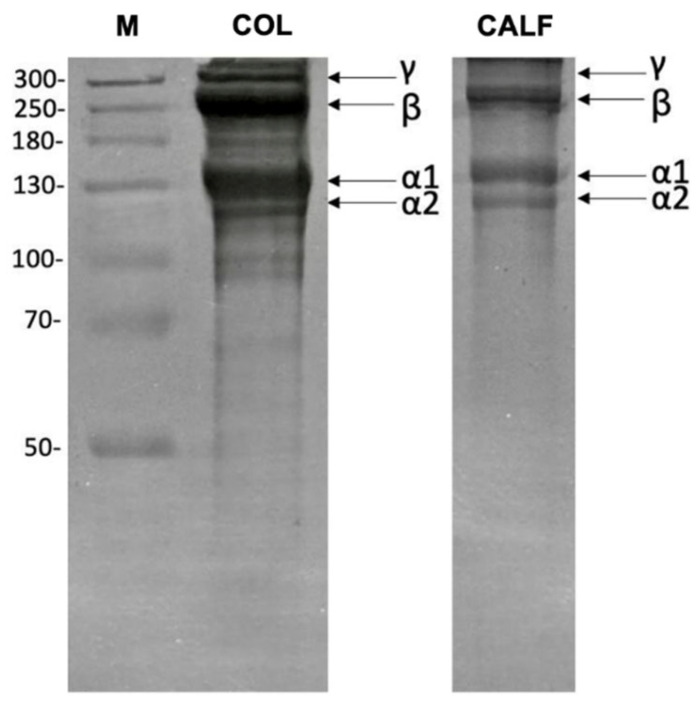
Sodium Dodecyl Sulphate—PolyAcrylamide Gel Electrophoresis (SDS–PAGE) (7.5%). Electrophoretic pattern for acid-soluble collagen (ASC); M: molecular weight marker, COL: sea bass skin collagen, CALF: calf skin collagen, type I.

The acid-soluble collagen (ASC) extraction by acetic acid is one of the most used methods to obtain collagen from marine organisms [35]. The collagen yield is dependent on the concentration of the solvent used up to a maximum concentration of 0.6 M, since higher solvent concentrations reduce collagen yield [36]. In this study, CH_3_COOH at the concentration of 0.5 M in a ratio of 1:50 (*w*/*v*) was used for the ASC extraction from *D. labrax* skin. The final yield of the extracted COL was 17.8% (dry weight basis), higher than that reported for collagen extracted from the European sea bass bones (approx. 4%). In fact, the collagen content in fish by-products can greatly differ depending on factors such as age, size, tissue, and species [37]. Type I collagen is the most common type of collagen in nature, found primarily in the vertebrate skin, consisting of a triple helix structure stabilized by interchain hydrogen bonds between glycine and amide groups in the polypeptide chains [38]. Sodium Dodecyl Sulphate—PolyAcrylamide Gel Electrophoresis (SDS-PAGE) analysis (Figure 2) showed characteristic bands of type I collagen, formed by an heterotrimer of two α_1_ chains and one α_2_ chain, with a molecular weight of approximately 135 kDa and 120 kDa, respectively, corresponding to the molecular mass previously reported for fish skin collagen [39]. Bands at higher molecular weights were also detected, with the β band at around 250 kDa and the γ band above 300 kDa, consistent with the presence of dimers (α_1_ and α_2_ chains together or two α chains—β chain) and trimers (3 α chains—γ chain), respectively [40]. On the contrary, lower molecular weight bands generally indicate degradation products.

### 2.2. Scaffolds Preparation and Characterization

In this study, 3D CS/COL scaffolds were successfully prepared by using a freeze-drying process, as described in paragraph 3.3. Two concentrations of CS (20–40 mg/mL) and COL (5–10 mg/mL) were considered.

**Figure 3:**
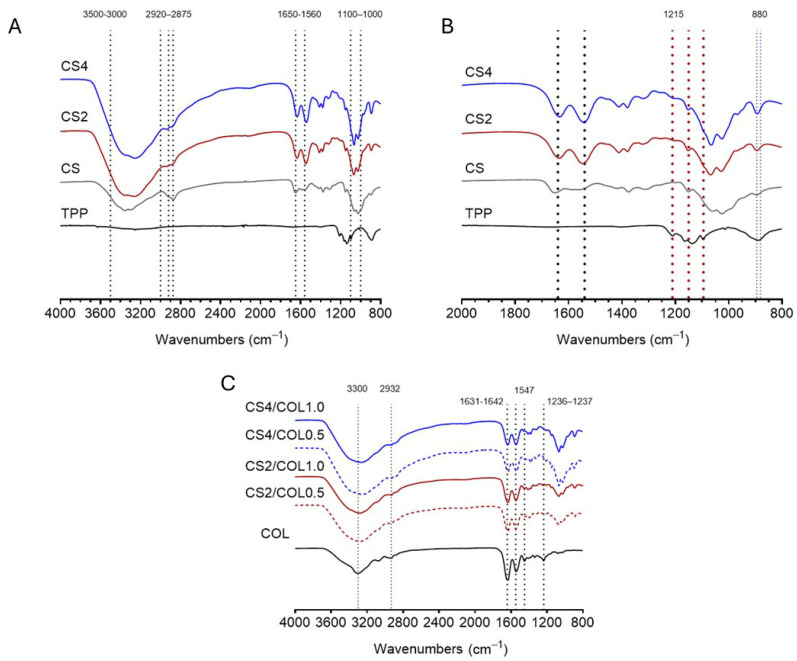
Attenuated total reflection Fourier-transform infrared (ATR-FTIR) of sodium triphosphate (TPP), chitosan scaffolds at 2% *w/v* and 4% *w/v* crosslinked with TPP (CS2 and CS4, respectively) and CS uncrosslinked (CS) between (**A**) 4000 and 800 cm^−1^, (**B**) 2000 and 800 cm^−1^ and (**C**) Collagen (COL), CS2 and CS4 with sea bass collagen (COL) (5 and 10 mg/mL) between 4000 and 800 cm^−1^.

Figure 3A,B show the effect of TPP crosslinking on CS scaffolds. In particular, the characteristic bands of CS were represented in Attenuated total reflection Fourier-transform infrared (ATR-FTIR) spectra. The bands between 3500 and 3000 cm^−1^ correspond to –OH and –NH stretching frequencies. It is possible to observe the bands of C–H stretching in the range 2920–2875 cm^−1^; C=O stretching of acetylate groups (Amide I) at 1650 cm^−1^; N–H bending of primary amine at 1550 cm^−1^ (Amide II). Meanwhile, the spectrum of TPP is characterized by the bands centered at 880 cm^−1^ attributed to the antisymmetric stretching of the P–O–P bond. The band centered at 1095 cm^−1^ can be attributed to the symmetric and antisymmetric stretching of the PO_3_ group, the one at 1150 cm^−1^ is related to the symmetric and antisymmetric stretching of the –PO_2_ group, and the one at 1215 cm^−1^ is related to the P=O stretching [41]. By comparing the spectra of CS2 and CS4 scaffold crosslinked with TPP with that of the pristine CS, several variations can be noticed due to the crosslinking reaction. Indeed, in the spectra of the modified scaffolds, besides the peak at 880 cm^−1^ typical of TPP molecule, a new band at 1215 cm^−1^, related to the antisymmetric stretching vibrations of –PO_2_ groups of TPP ions, is present as an index of the formation of ionic interactions between CS and TPP. This latter peak was not very pronounced in the spectrum of sample CS2 compared to CS4, probably due to a lower density of crosslinking binds (lower CS concentration) [42,43].

The presence of COL into the CS polymer blend was confirmed by the ATR-FTIR spectra reported in Figure 3C. The Amide A position of COL was found at 3300 cm^−1^, which represents the absorption band of N–H stretching, and indicates the presence of hydrogen bonds. The amide B band of COL was observed at 2932 cm^−1^, which is related to the CH_2_ asymmetric stretch. The peak of Amide I also associated with the stretching vibrations of the carbonyl groups (C=O bond) along the polypeptide backbone was observed at 1631–1642 cm^−1^. Meanwhile, the Amide II band of CS/COL appeared at 1547 cm^−1^. Finally, the Amide III band resulting from NH bend coupled with CN stretch and NH bend was observed at 1236–1237 cm^−1^. Table 1 summarizes the characteristic ATR-FTIR bands together with their assignments to functional-group vibrations.

The ATR-FTIR spectra of the CS2/COL and CS4/COL scaffolds exhibited distinct features that reflect their compositional differences, specifically the lower chitosan content in CS2 relative to CS4. Furthermore, the presence of lower or higher COL content (COL0.5 vs. COL1.0) seemed also to influence the relative amide band intensities. Generally, CS4/COL showed a stronger, broader band at ~3300 cm^−1^ than CS2/COL. This evidence may be ascribed to a higher chitosan content (CS4) that supplies more –OH/–NH groups and/or stronger hydrogen-bonding networks with collagen, producing a larger, broadened stretch. The C–H stretching (≈2932 cm^−1^) appeared more pronounced in the CS4 spectra than in CS2, probably due to a more polymer (chitosan) backbone signal when chitosan concentration increases. The intensity of Amide I (≈1631–1642 cm^−1^) and Amide II (≈1547 cm^−1^) changed between CS2 and CS4 samples and also with COL0.5 compared to COL1.0. CS4/COL tended to show broader or slightly shifted peaks compared with CS2/COL. Indeed, by increasing CS concentration, the local environment of COL peptide bonds (electrostatic interactions and hydrogen bonding) was altered. This could shift or broaden the amide bands. Furthermore, higher collagen loading (COL1.0) increased absolute amide intensities, but the presence of more chitosan modified those band shapes. Finally, regarding Amide III/fingerprint region (≈1236–1237 cm^−1^ and nearby), differences are also visible: CS4/COL spectra showed changes in intensity/structure relative to CS2/COL, suggesting different degrees of interaction between CS and COL and different relative contributions from each polymer in that region.

Moreover, the ATR-FTIR data, showing increased amide I/II intensities and broadened OH/NH stretches in the higher-collagen scaffolds, are consistent with molecular dynamics (MD) simulations by Przybyłek et al., which report that a high deacetylation degree (DD) in CS and a high hydroxylation degree of proline moieties in collagen could lead to favorable binding free energy [44]. This supports the interpretation that higher COL loading with higher available amine groups leads to a network strongly reinforced by intermolecular hydrogen bonding.

**Figure 4:**
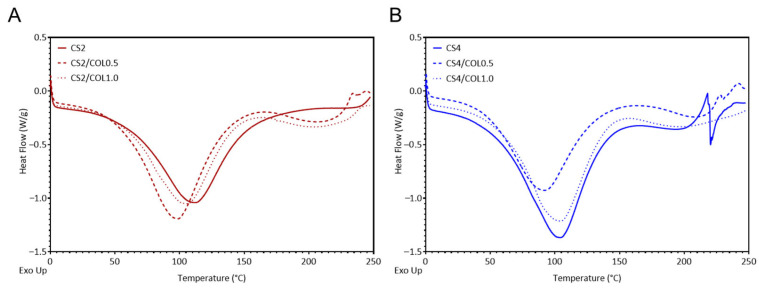
Differential scanning calorimetry (DSC) thermogram of CS and CS/COL scaffolds. (**A**) CS2, CS2/COL0.5 and CS2/COL1.0, (**B**) CS4, CS4/COL0.5 and CS4/COL1.0.

To better understand structural features, differential scanning calorimetry (DSC) was performed. Results, reported in Figure 4, showed that the temperature of maximum dehydration (T_d_) was sensitive to both CS and COL concentrations. Neat CS2 (111 °C) retained water more strongly than neat CS4 (105 °C). Addition of a low COL amount (COL0.5) lowered T_d_ in both groups (CS2/COL0.5 = 99 °C; CS4/COL0.5 = 92 °C), whereas increasing collagen to COL1.0 restored T_d_ toward the values of the corresponding neat chitosan (CS2/COL1.0 = 105 °C; CS4/COL1.0 = 104 °C). These trends indicate that small amounts of COL may increase thermal stability and the fraction of loosely bound or free water, while higher COL content may promote stronger intermolecular hydrogen-bond networks with CS that retain water more tightly. Furthermore, in the CS2 scaffolds, where CS network was less concentrated, the addition of a small amount of collagen (COL0.5) produced the largest enthalpic response, indicating that CS/COL hydrogen bonding enhanced structural cooperativity and strengthened the thermal transition. At higher collagen content (COL1.0), enthalpy decreased, suggesting that excess COL may introduce heterogeneity and disrupt large cooperative domains. By contrast, in the CS4 groups, where the CS matrix was already more tightly packed, the addition of COL lead to a progressive reduction in enthalpy despite the consistent increase in transition temperature. This may indicate that COL embedding at higher CS concentration may stabilize the system through additional intermolecular interactions but simultaneously may interfere with CS-CS cooperative domains, yielding a less cooperative transition. Taken together, these findings demonstrate that the balance between stabilization and cooperativity is strongly dependent on the CS/COL ratio: at low CS concentration COL acts as a synergistic local plasticizer, whereas at high CS concentration COL primarily modifies the network architecture, enhancing stability but reducing cooperativity. The results are in line with previous works [45,46].

**Figure 5:**
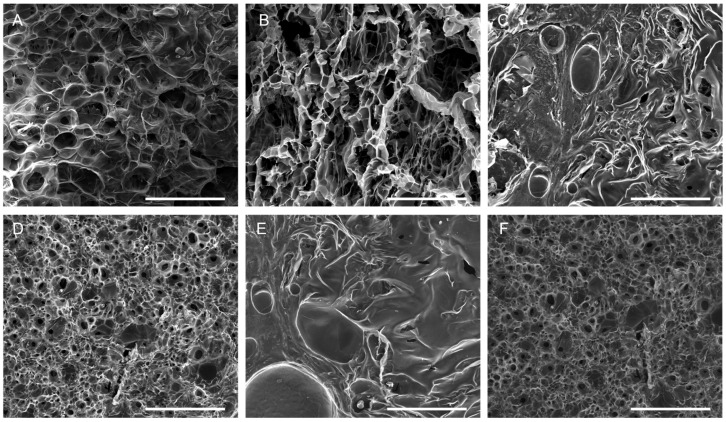
Morphological analysis of scaffolds. Scanning electron microscopy performed on (**A**) CS2, (**B**) CS2/COL0.5, (**C**) CS2/COL1.0, (**D**) CS4, (**E**) CS4/COL0.5 and (**F**) CS4/COL1.0. (Magnification (Mag.) 200×, scale bar 500 μm).

Since the scaffolds are intended for application on wound surfaces post-lyophilization, the morphology of the 3D CS and CS/COL scaffolds was evaluated after freeze-drying. All scaffold types revealed an open porous architecture with a high degree of interconnectivity, according to scanning electron microscopy (SEM) images (Figure 5). This structural characteristic is crucial for wound healing because it creates a favorable *milieu* that promotes cell adhesion, migration, and proliferation. CS2 showed large and irregular pores with thin pore walls. The structure was highly open, but with less structural integrity, consistent with a lower polymer concentration (Figure 5A). CS2/COL0.5 highlighted a more organized pore structure compared to neat CS2, with collagen fibrils integrated within the matrix, reinforcing pore walls. The structure appeared relatively porous, but with better wall definition and slightly denser architecture (Figure 5B). By increasing COL concentration (CS2/COL1.0), the scaffold appeared much denser with pores partly filled by collagen. It is possible to observe thickened walls and a reduced porosity that could contribute to greater mechanical stability (Figure 5C). CS4 scaffolds showed small and uniform pores with dense and compact walls, suggesting that a higher chitosan concentration may lead to a tighter network (Figure 5D). In the presence of COL (Figure 5E,F), it is possible to observe a dense matrix, but with visible large collagen fibril bundles reinforcing the structure (CS4/COL0.5). Furthermore, COL partially interrupts the compactness, giving larger pore-like features. For this reason, pore interconnectivity appeared still limited compared to CS2/COL0.5. When COL concentration increased (CS4/COL1.0) a dense and uniform porous network with thick pore walls was obtained. COL seemed more homogeneously distributed than in CS4/COL0.5, leading to a reinforced but compact structure. The overall porosity was the lowest among the groups but probably leading to higher mechanical strength. In conclusion, looking at CS2 scaffold (Figure 5A–C), larger pores, higher porosity were obtained. COL gradually stabilized the structure. Meanwhile, for CS4 scaffolds (Figure 5D–F), smaller and denser pores were visible. COL addition modified compactness with CS/COL0.5 showing visible collagen bundles and CS4/COL1.0 resulting in a more homogeneous dense network.

The SEM confirmed the FTIR interpretation. Indeed, in CS4/COL, stronger chitosan–collagen interactions, shown by amide band shifts and broad OH/NH stretching, led to denser scaffolds. In contrast, CS2/COL scaffolds retained higher porosity, with collagen contributing more visibly to pore wall reinforcement. The same trend was reported in the literature [42,47]. In its natural state, the collagen triple helix is stabilized by direct chemical bonds, hydrogen bonds, and water-bridged crosslinks. When blended with CS, the abundant amino groups in its molecular chains not only reinforce collagen fibers by anchoring them in place but also act as effective crosslinking agents.

**Figure 6:**
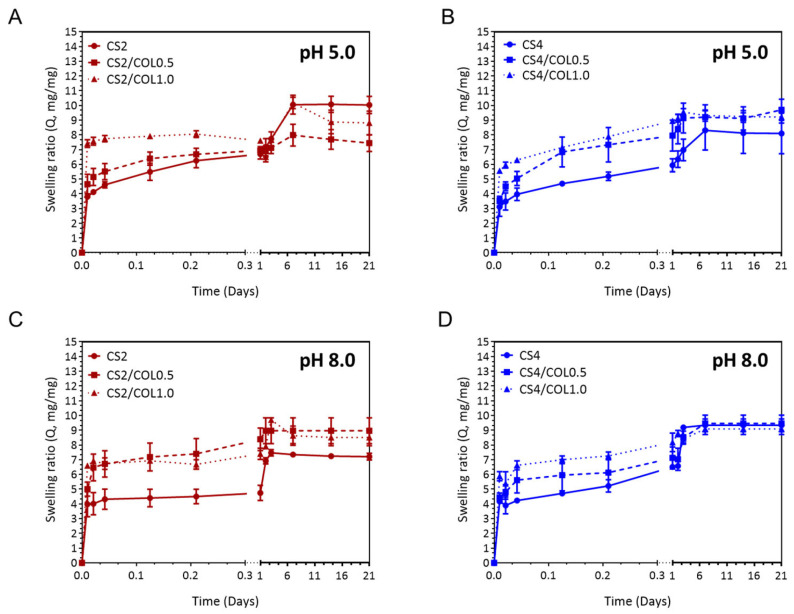
Swelling behavior of CS and CS/COL scaffolds over time performed in PBS up to 21 days at (**A**,**B**) pH 5.0 and (**C**,**D**) pH 8.0. Q is expressed as mg/mg ± S.E.M.

Effective wound dressings must be capable of absorbing exudate while preserving a moist environment, which is vital for optimal wound care. Additionally, dressings with higher swelling capacity and prolonged water retention can maintain a humid environment that protects newly formed granulation tissue and promotes faster surface cell migration, thereby reducing the risk of scarring [48]. To achieve this, the swelling behavior of the dressings was carefully examined. Figure 6 reports the swelling ability of 3D neat and CS/COL scaffolds at pH 5.0 (Figure 6A,B) and pH 8.0 (Figure 6C,D). All scaffolds showed rapid swelling in the first hours, as expected for porous polymeric hydrogels. At pH 5.0, by increasing the CS concentration, the swelling ratio Q slightly decreased; meanwhile, at pH 8.0, an opposite trend was observed. Regarding CS2-based scaffolds, at pH 5.0, the addition of COL reduced equilibrium swelling progressively (CS2/COL1.0 < CS2/COL0.5 < CS2) (Figure 6A). This result is also in agreement with SEM images, where the COL partially filled and reinforced pore walls, consequently reducing free pore volume and limiting water penetration compared with neat CS2 (Figure 5A–C). Meanwhile, for CS4-based scaffolds, adding a moderate amount of collagen (CS4/COL0.5) increased swelling relative to CS4 alone; a further addition (CS4/COL1.0) reduced it again. This result is in line with SEM images, where it is possible to observe that COL0.5 created microvoids and additional hydrophilic domains leading to an increased swelling ratio. Meanwhile, a higher COL loading (COL1.0) filled the limited pore space rather than creating accessible microvoids, producing a compact blend that absorbed a lower amount of water (Figure 5D–F). However, the swelling of CS4/COL1.0 often remained ≥ CS4 alone (Figure 6B).

Although CS2-based scaffolds still swelled more than CS4-based ones overall, COL helped to close the gap by suppressing CS2 swelling and, at moderate loading, enhancing CS4 swelling. At pH 8.0, the equilibrium swelling of CS2 was reduced compared to pH 5.0; collagen still reduced swelling (CS2/COL1.0 < CS2/COL0.5 < CS2), but the absolute differences were smaller because CS was less ionized (Figure 6C). CS4 showed lower swelling at pH 8.0; the collagen-induced increase detected for CS4/COL0.5 at pH 5.0 appeared more attenuated or disappeared at pH 8.0. High collagen (CS4/COL1.0) generally produced a denser scaffold and so the lowest swelling (Figure 6D). It is worth noting that CS2/COL still tended to have higher water uptake than CS4/COL, but both were substantially lower than at pH 5.0 and the collagen-driven modulation resulted weaker.

However, at acidic pH, Q was always slightly higher for all the compositions (Figure 6C,D). This different behavior could be ascribed to the known dissolution phenomenon of CS, being the pH lower than its pKa (≈6.3–6.5) [49]. The results obtained in the present work are in line with others in the literature [41,48].

In conclusion, in CS2-based scaffolds characterized by a lower polymer concentration, COL acted mainly as a structural filler/reinforcement. Adding COL reduced free pore volume and increased crosslink-like hydrogen bonding with CS leading to decreased swelling. This effect was evident at both pH values, but more marked at pH 5.0 because baseline swelling was higher. In CS4-based scaffolds, instead, the dense CS network initially restricted water uptake. A moderate collagen addition (CS4/COL0.5) probably altered the compact CS4 network, creating additional hydrophilic domains and microvoids, thus swelling increased compared to CS4 alone. This effect was mostly pronounced at pH 5.0. Higher collagen (CS4/COL1.0) tended to re-densify the matrix, with more fibrils filling pores and more H-bonding, then reducing swelling again. The results also showed that pH moderated CS/COL interactions. At pH 5.0, protonated CS could interact electrostatically and via H-bonding with COL, producing stronger network changes and hence a larger modulation of swelling. At pH 8.0 these electrostatic contributions were absent/reduced, so the collagen effect was dominated by steric/fibrillar filling and hydrogen bonding, leading to overall smaller changes in swelling. MD simulations also predict that while affinity (binding free energy) decreases with increasing pH due to reduced protonation of CS amino groups, all complexes remain thermodynamically stable across a broad pH range [50]. This explains why in pH 8.0 swelling experiments, even though swelling is lower, scaffolds with collagen still retain structure and show reduced degradation relative to pure neat controls.

**Figure 7:**
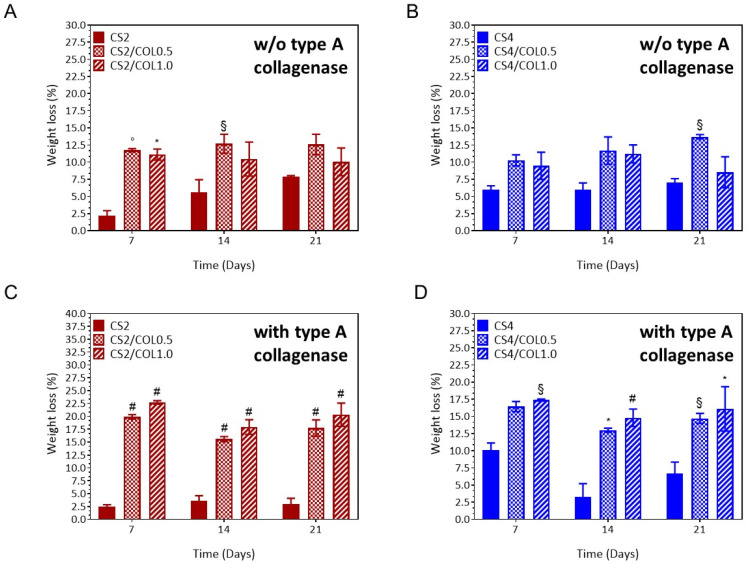
Results from stability test. Weight loss (expressed as % ± S.E.M.) of CS/COL scaffolds in phenol red-free Dulbecco’s Modified Eagle Medium low glucose supplied with antibiotics, (**A**,**B**) without (**C**,**D**) and with type A collagenase (1 U/mL). Statistical analysis of variance of the means was assessed by *two-way* ANOVA and Bonferroni post hoc test (^§^ *p* < 0.1; * *p* < 0.01; ◦ *p <* 0.001, ^#^ *p <* 0.0001), by considering as control neat CS scaffold for each concentration at each time.

All the scaffolds maintained their 3D structure without collapsing over time, suggesting their suitability for skin tissue engineering [51]. Specifically, the scaffolds should not completely degrade before the wound healing process’s proliferation phase, which typically lasts two to four weeks, is finished [52]. In the present study, neat CS2 scaffolds showed a weight loss (%) in the range 2.2–7.9%, over the time period (Figure 7A). By increasing the COL concentration, CS2-based scaffolds showed a significant higher weight loss (10.1–12.7%), compared to CS alone [CS2/COL0.5 vs. CS, ◦ *p <* 0.001 (7 days); CS2/COL1.0 vs. CS2, * *p* < 0.01 (7 days) and CS2/COL0.5 vs. CS2, ^§^ *p* < 0.1 (14 days)], without significant differences between CS2/COL0.5 and CS2/COL1.0 (Figure 7A). This result was clearly expected as CS2 is more resistant to degradation than collagen because of its higher crystallinity and stronger polymer backbone. On 21 days, there were no differences among the groups. A similar trend was observed for CS4-based scaffolds, even though the effect of collagen addition was less pronounced than in CS2 (Figure 7B). Higher CS content provided stronger structural reinforcement, limiting degradation despite collagen incorporation (Figure 7B). Moreover, the higher amount of CS4 compared to CS2 allowed stabilizing the CS/COL blend that did not show significant differences among the groups at short times. Moreover, CS/COL scaffolds were incubated in Type A collagenase solutions (1 U/mL) to assess the enzymatic degradation profile (Figure 7C,D). It was hypothesized that the constant degradation rate was due to the presence of matrix metalloproteinases-sensitive motifs in COL biomaterials, which are specific amino acid sequences that are recognized and cleaved by enzymes involved in ECM remodeling [48]. Herein, results highlighted that in presence of collagenase, degradation was dramatically accelerated in collagen-containing scaffolds (COL0.5 and COL1.0), with weight loss reaching ~30–35% by day 21, compared to <10% for pure CS2 (Figure 7C). Since collagen is the specific target of collagenase, higher collagen content makes the scaffold more vulnerable. In particular, CS2/COL scaffolds had values of weight loss (%) between 17.7% (CS2/COL0.5) and 20.3% (CS2/COL1.0), higher than CS2 (3.0%,CS2/CO0.5 and CS2/COL1.0 vs. CS2, ^#^*p <* 0.0001, 21 days). Meanwhile slightly lower values were detected for CS4/COL scaffolds; 14.7% (CS4/COL0.5) and 16.9% (CS4/COL1.0), thus suggesting a protection effect of COL exerted by CS (Figure 7D).

In conclusion, the addition of collagen allowed to tune the degradation behavior of CS that is resistant to hydrolytic and enzymatic degradation. In general, collagen-rich scaffolds degraded faster even without enzymes, due to increased hydrophilicity. This effect was more pronounced in CS2 scaffolds, which, owing to their lower CS content, showed significantly faster weight loss, whereas CS4 scaffolds retained greater stability over time.

Ultimately, achieving an optimal balance between the physicochemical properties (such as swelling capacity and degradation rate) and mechanical characteristics is essential. As wound dressings or scaffolds, the materials must exhibit sufficient mechanical strength to resist external stresses while preserving their structural stability. It is well known that native skin has a Young’s modulus between 10 and 50 kPa. Research has shown that tissue regeneration is more effective when scaffold stiffness aligns with that of host dermal tissue, approximately 50 kPa. Additionally, the scaffold should retain its integrity throughout all phases of the tissue remodeling process [53].

**Figure 8:**
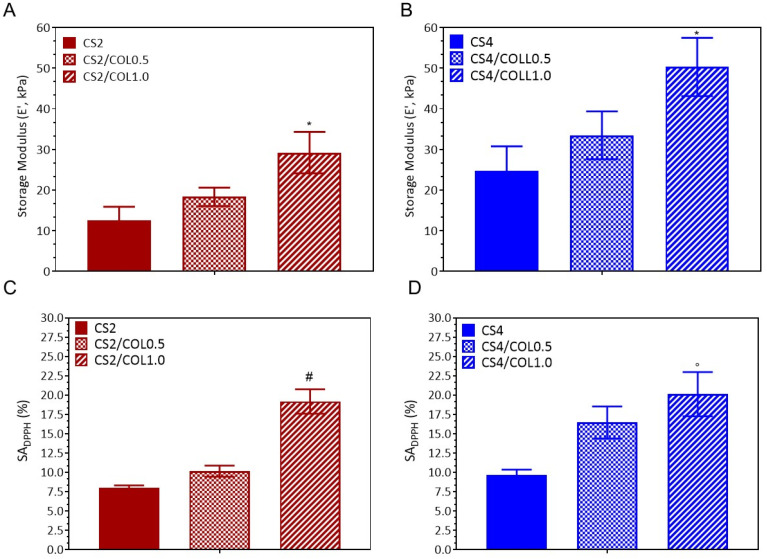
(**A**,**B**) Dynamic mechanical analysis (DMA) performed on CS2 and CS4 scaffolds with and without collagen (5 and 10 mg/mL). Results are expressed as mean value of the storage modulus ± standard deviation. (**C**,**D**) The 2,2-diphenyl-1-picrylhydrazy (DPPH) scavenging activity of CS/COL scaffolds. Data are expressed as % mean value of DPPH scavenging activity (SA_DPPH_ ± S.E.M.). Statistical analysis of variance of the means was assessed by *one-way* ANOVA and Bonferroni post hoc test (* *p* < 0.01; ◦ *p <* 0.001, ^#^ *p <* 0.0001), by considering as control neat CS scaffold for each concentration.

Dynamic mechanical analysis (DMA) results showed that the storage modulus is strongly influenced by both CS and COL concentrations (Figure 8A,B). Neat CS2 showed the lowest modulus (12.6 kPa). Collagen reinforced the weak CS2 matrix, but only at higher concentration the effect became strong enough to reach statistical significance. Indeed, CS2/COL0.5 was characterized by a storage modulus of 18.3 kPa, which was not significant different vs. CS2; meanwhile, CS2/COL1.0 showed a value of 29.2 kPa (* *p* < 0.01, vs. CS2) (Figure 8A). Regarding CS4 scaffolds, CS4 already showed a higher modulus (27.1 kPa). The addition of COL further enhanced stiffness: CS4/COL0.5 (31.9 kPa, not significant, vs. CS4) and CS4/COL1.0 (51.3 kPa, significant, ◦ *p* < 0.001, vs. CS4) (Figure 8B). Such high elastic moduli were useful considering the significant forces occurring during wound healing [54] and they have also been demonstrated to favorably stimulate fibroblast growth [55].

When it comes to wound applications, a scaffold’s inherent antioxidant features are particularly crucial. In fact, during the initial phases of inflammation and microbial attack, scaffolds may regulate and limit the formation of reactive oxygen species (ROS). Herein, the antioxidant properties of scaffolds were studied by 2,2-diphenyl-1-picrylhydrazy (DPPH) test (Figure 8C,D). CS/COL0.5 and CS/COL1.0 scaffolds showed DPPH scavenging activity values generally higher than their respective control. This can be ascribed to the presence of aromatic compounds (such as tyrosine and phenylalanine) of COL [56]. By increasing CS concentration, values of SA_DPPH_ slightly increased. Indeed, CS’ antioxidant activity is mainly attributed to its free amino and hydroxyl groups, which can scavenge free radicals [57].

### 2.3. In Vitro Biological Evaluation of Scaffolds

**Figure 9:**
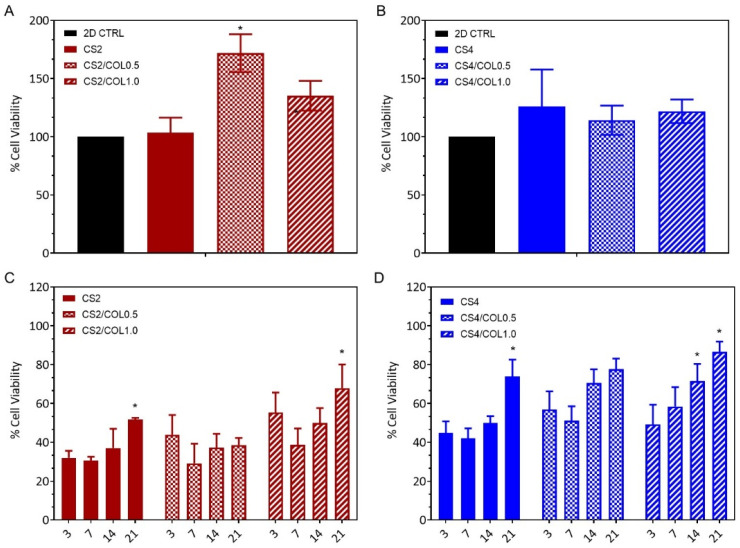
Evaluation of the cellular toxicity of scaffolds on murine fibroblast cell line. Indirect in vitro cytotoxic assay on L929 cell line of (**A**) CS2 and CS2/COL; (**B**) CS4 and CS4/COL scaffolds. Results are expressed as percentage of cell viability after 24 h of exposure (n = 3). Statistical analysis of variance of the means was assessed by ANOVA and post hoc test (* *p* < 0.01), by considering 2D CTRL as control. Cell viability of L929 fibroblasts cultured on (**C**) CS2 and CS2/COL; (**D**) CS4 and CS4/COL scaffolds. Cell viability was assessed at time 0 and at 3, 7, 14, and 21 days, and expressed as the percentage of viable cells relative to the control (cells attached at time 0). Data are presented as mean ± standard deviation (n = 3). Statistical analysis of variance of the means was assessed by Student’s *t*-test (* *p* < 0.05), by comparing viability at days 7, 14, and 21 with day 3.

The potential indirect cytotoxic effects of the scaffolds were evaluated using murine L929 fibroblasts. Fibroblasts are a widely used cell model for in vitro cytotoxicity and biocompatibility studies, since they play a crucial role in tissue repair and producing/remodeling ECM [58,59]. For this purpose, 100 mg of each scaffold was soaked in 2.5 mL low-glucose DMEM overnight to allow the possible release of any cytotoxic components into the medium. Then, the resulting supernatants (conditioned media) were used as media for cell growth. As a negative control, cells were cultured in unconditioned low-glucose DMEM. As shown in Figure 9A,B, after 24 h of treatment, the conditioned media did not show any cytotoxic effect on L929 cells compared to the control. On the contrary, cells treated with media conditioned with scaffold CS2/COL0.5, CS2/COL1.0, CS4, and CS4/COL1.0 showed an increase in growth up to 175%, compared to the control. This result indicates that these scaffolds did not interfere with cell viability and proliferation and could potentially promote a pro-proliferative activity.

The viability of L929 fibroblasts grown onto scaffolds was evaluated over a 21-day period using the MTT assay, to assess which scaffold composition could better promote cell growth and proliferation. As shown in Figure 9C,D, all scaffolds supported cell attachment and growth, with a variable degree. A common trend observed for all scaffolds was characterized by a low cell viability in the early time points (3 and 7 days), with increasing values after 14 and 21 days.

Among the groups, scaffolds CS4/COL1.0 demonstrated a steady increase in cell viability over time (Figure 9D), showing the highest percentage of viable cells on day 21 (87%). This scaffold exhibited a clear biocompatibility, showing a proliferation trend from day 3 to day 21.

This result could probably be ascribed to the presence of the highest COL concentration combined also with the improved mechanical properties and the enhanced structural stability of the scaffolds. Indeed, as shown by the results in the previous paragraph, Young’s modulus of the scaffold CS4 produced with marine collagen increased up to 51.3 kPa, a value that positively enhances fibroblast attachment and proliferation [55], indicating strong applicability in tissue regeneration.

Scaffolds CS2 and CS2/COL1.0 also supported cell viability across time points but at lower levels (Figure 9C). Notably, CS2/COL1.0 showed a moderate improvement in cell viability, reaching nearly 70% on day 21. Scaffolds CS4/COL0.5 and CS2/COL0.5 did not show a significant increase in cell viability with respect to day 3.

These results suggest that marine collagen improves the fibroblast attachment and proliferation into a 3D scaffold; this behavior is in accordance with previous findings, where cells [60] and in vivo models [61] showed an enhancement in growth rate and tissue repair when marine collagen was used as part of scaffold composition. This could suggest that the use of marine collagen may provide an adequate porosity and a beneficial environment for cell adhesion and growth, potentially improving the overall effectiveness of the scaffold for tissue growth or repair applications.

**Figure 10:**
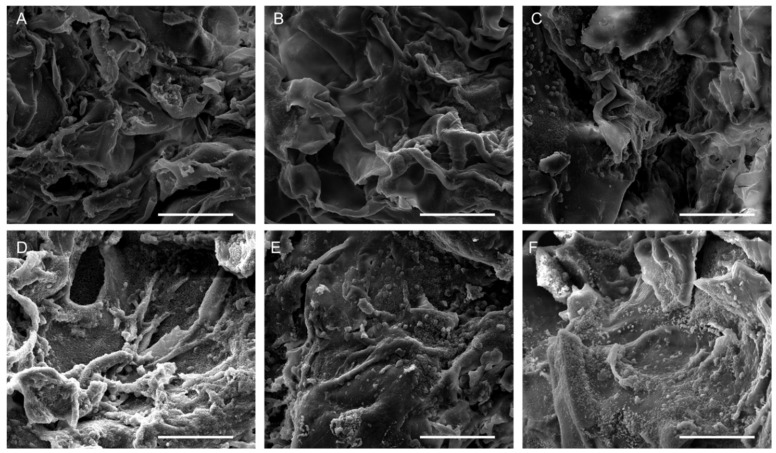
Morphological analysis of scaffolds after fibroblast attachment and proliferation. SEM analysis performed on (**A**) CS2, (**B**) CS2/COL0.5, (**C**) CS2/COL1.0, (**D**) CS4, (**E**) CS4/COL0.5 and (**F**) CS4/COL1.0 cellularized with L929 fibroblast cells seeded on the top of the scaffolds. [Magnification (Mag.) 800×, scale bar 100 μm].

After 21 days of incubation with L929 cells, the morphological analysis still showed a highly porous and complex architecture (Figure 10), that in turn may facilitate fundamental cell processes such as cell migration, nutrient diffusion, and catabolites removal, favoring cell attachment and proliferation [62]. Particularly, SEM images showed significant cell attachment and spreading across both the scaffold surfaces, CS2-based (Figure 10A–C) and CS4-based ones (Figure 10D–F). However, even though cells appeared well-integrated into the scaffold matrix, covering scaffold surfaces with high density and extending cytoplasmic projections in both cases, on CS4/COL1.0 cells appeared elongated, closely clustered, and adherent to one another, indicating the induction of tissue formation. This indicates active interaction between cells and substrates. These findings confirm viability results were the 3D scaffold supported long-term (up to 21 days) cell growth and proliferation. Similarly, in previous studies, scaffolds composed of chitosan and collagen of marine origin (e.g., sponges) were found not to be toxic and able to support 3D cell growth [63,64].

Thus, our biological results can contribute to better optimizing the combination of marine collagen and chitosan to create a favorable 3D microenvironment for fibroblast growth. This can probably trigger specific intracellular signaling pathways, which should be investigated in the future. Indeed, data already reported showed the activation of key proliferative factors (i.e., epidermal growth factor, fibroblast growth factor, vascular endothelial growth factor) in murine cells cultured on marine collagen derived from fish skin [65].

### 2.4. Antimicrobial Activities

**Figure 11:**
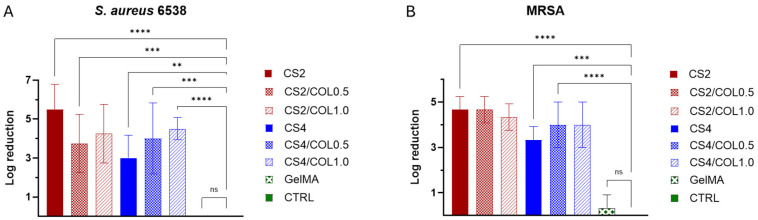
Antimicrobial activity of the developed scaffolds. Antimicrobial efficacy is expressed as Log reduction of *S. aureus* 6538 (**A**) and MRSA (**B**), calculated relative to the control (CTRL). Data are presented as the mean ± standard deviation (SD). Statistical analyses were determined by ANOVA with Dunnett’s test for multiple comparisons. Significances are referred to the CTRL. **** *p* < 0.0001, *** *p* < 0.0002, ** *p* < 0.0021, ns (not statistically significant).

*S. aureus* strains, in particular MRSA, represent the leading agents in skin infections, especially in wound infections and in the chronicization of infected wounds [66]. Therefore, it is crucial to evaluate whether the developed CS/COL scaffolds effectively inhibit the growth of *S. aureus* to validate their potential as a functional biomaterial for skin healing. The antimicrobial activity of the scaffolds was evaluated against *S. aureus* ATCC 6538 and MRSA. After overnight incubation, in absence of scaffold, both bacteria showed robust bacterial growth, reaching a density of 11.4 Log (CFU/mL). Moreover, GelMA, the negative control, showed a similarly high concentration (11.6 Log (CFU/mL)) and a non-significant log reduction (Figure 11A,B), confirming its non-inhibitory nature. All the evaluated scaffolds had a statistically significant effect (*p* < 0.05) on bacterial viability compared to the control and exhibited a bactericidal effect, with a Log reduction greater than 3 (a reduction of 99.9% or more in bacterial growth). CS2 scaffold was the most active, reducing the bacterial count to 6.1 Log (CFU/mL) and 6.9 Log (CFU/mL) for *S. aureus* 6538 and MRSA, respectively. This corresponded to the highest log reduction of 5.4 and 4.6 (Figure 11B). CS4 scaffold was less active for both strains, yielding a log reduction of 3.0 and 3.2 for *S. aureus* 6538 and MRSA, respectively. The antimicrobial activity of the chitosan-containing scaffolds was an expected outcome, as chitosan is widely reported for its antimicrobial activity [67]. However, the exact mechanism of action is unknown, and various hypotheses have been proposed. The major believed mechanism is based on a proposed electrostatic interaction of positively charged NH_3_^+^ groups of chitosan with the negatively charged components of bacterial cell membranes. This interaction could increase the membrane permeability, which may cause osmotic imbalances or hydrolysis of peptidoglycan, causing leakage of intracellular contents. Another proposed mechanism is the chitosan binding to microbial mRNA, preventing protein synthesis. Additionally, chitosan could penetrate the nucleus and directly interact with microbial nucleic acids [68,69].

A clear antimicrobial activity was still observed when collagen was incorporated into the CS-based scaffolds, suggesting that the addition of marine collagen maintains the already known antimicrobial activity of chitosan (Figure 11).

## 3. Materials and Methods

### 3.1. Sea Bass Skin Preparation and Acid Soluble Collagen (ASC) Extraction

Sea bass by-products were purchased from local markets in Naples, Italy. Fish skin was washed with cold water, descaled, cut into small pieces (about 1 cm^2^), air dried for 3 h, and then used for extraction.

ASC was extracted from fish skin samples according to the method described by Kittiphattanabawon and collaborators [70], with some modifications. All procedures were carried out at 4 °C. Initially, the fish skin was subjected to two different pre-treatment steps. In particular, to remove non-collagenous proteins, fish skin was soaked in 0.1 M sodium hydroxide (NaOH, Sigma Aldrich, Milan, Italy) with a ratio of 1:50 (*w*/*v*) for 24 h, twice. Then, the skin was washed with water to reach a neutral pH. To remove lipids, the skin was soaked in 10% isopropyl alcohol (IPS, Honeywell, Germany), with a skin/solution ratio of 1:10 (*w*/*v*) for 2 days, changing the solution every day. Then, the skin was washed with water until reaching neutral pH and used for the extraction of collagen by using 0.5 M acetic acid (CH_3_COOH, Biochem Chemopharma, Cosne sur Loire, France), with a ratio of 1:50 (*w*/*v*) for 3 days, twice.

The insoluble components were separated with cotton cloth, and the filtrate was centrifuged at 4 °C, 20,000× *g* for 1 h. The obtained supernatant was precipitated by adding sodium chloride (NaCl, Sigma Aldrich, Milan, Italy) to a final concentration of 2.6 M and Tris hydrochloric acid (HCl, Sigma Aldrich, Milan, Italy) 0.05 M. The precipitate containing collagen was collected by centrifuging at 4 °C, 20,000× *g* for 1 h. The pellet was dissolved at a minimum volume of 0.5 M CH_3_COOH, and dialyzed against 0.1 M CH_3_COOH, followed by distilled water. The dialysate was freeze-dried and subsequently stored at –20 °C. Collagen yield was calculated as:

(1) $$Collagen\;yield\left(\%\right)=\frac{Weight\;of\;freeze-dried\;collagen}{Weight\;of\;dried\;fish\;skin}\times 100$$

### 3.2. SDS-PAGE

SDS–PAGE was performed by the method of Laemmli [71] with modification [34]. 1 mg of COL was dissolved in 100 µL of CH_3_COOH 0.5 M, then mixed with 100 µL Tris-HCl, pH 8.8, containing 4% SDS. Solubilized samples were heated at 50 °C for 5 min and then mixed with the sample buffer 2× (0.5 M Tris–HCl, pH 6.8, 5% SDS, 20% glycerol, 10% 2-mercaptoethanol). Samples were loaded onto a polyacrylamide gel made of 7.5% separating gel and 4% stacking gel, and subjected to electrophoresis at a constant current of 120 V. The gel was stained with Coomassie Brilliant Blue, and the Spectra Multicolor High Range Protein Ladder (Thermo Fisher Scientific, Waltham, MA, USA) was used as Marker. Calf skin collagen (Sigma Aldrich, Burlington, MA, USA) was used as standard.

### 3.3. Chitosan/Collagen Scaffold Preparation

A controlled interconnected porous CS/COL scaffold was prepared by a freeze-drying process. Chitosan (CS, Sigma Aldrich, low molecular weight, M_w_ 50–190 kDa based on viscosity, degree of deacetylation ≥75%) and extracted COL were dissolved in 0.5 M CH_3_COOH. The slurry was mixed using a magnetic stirrer. The resulting suspension was dropped in a Petri dish plate (Ø = 90 mm) and frozen in a controlled way, by following an adapted protocol [52], 4 °C for 30 min, −20 °C for 1.5 h, and −80 °C overnight, then freeze-dried under vacuum in a SCANVAC COOLSAFE (Labogene Scandivian by Design freeze dryer—Bjarkesvej, Denmark). Two different concentrations of CS (20 and 40 mg/mL) and two concentrations of COL (5 and 10 mg/mL) were considered.

Neat and CS/COL cylindrical scaffolds (diameter (d) = 6 mm, height (h) = 4 mm) were obtained by cutting the membrane using biopsy punches and were soaked in 5 mg/mL sodium tripolyphosphate (TPP, Sigma Aldrich technical grade, 85%, Milan, Italy) for 6 h at 4 °C. After this period, scaffolds were rinsed in deionized water (dH_2_O, for chromatography LC-MS grade, conductance at 25 °C ≤ 1 μS/cm) to remove the excess of salt until pH 7.0 and, finally, submitted to a second step of freeze-drying. The nomenclature of the samples is reported in Table 2.

### 3.4. Scaffold Characterization

#### 3.4.1. Physicochemical Analysis

The composition and crosslinking of the polymer blends were evaluated using ATR-FTIR spectroscopy (Thermo Fisher Nicolet IS10, Waltham, MA, United States of America). The material was scanned at a resolution of 2 cm^−1^ between 800 and 4000 cm^−1^.

#### 3.4.2. Differential Scanning Calorimetry

A thermal analyzer Mettler DSC 822/400 (Mettler-Toledo, Columbus, OH, USA equipped with a DSC cell purged with nitrogen (50 mL/min) and chilled with liquid nitrogen. Scaffolds of about 5 mg were subsequently heated, cooled, and reheated at a rate of 10 °C/min from 0 to 250 °C.

#### 3.4.3. Morphological Analysis

The morphology of 3D scaffolds was investigated by SEM (FEI Quanta 200 FEG, Hillsboro, OR, United States of America). An ion sputter was used to apply an ultrathin layer of Au/Pt to lyophilized samples before observation.

Cells’ morphology and spreading after being seeded on 3D scaffolds were evaluated by SEM analysis. For this aim 8 × 10^4^ cell/scaffold were seeded and grown for 21 days at 37 °C. After these time points, cells were fixed with a solution of 4% *v/v* paraformaldehyde (Sigma Aldrich, Milan, Italy) for 3 h, at 4 °C, washed with phosphate-buffer solution (PBS, Sigma Aldrich, Milan, Italy), (1×, pH = 7.4) and dehydrated using a series of increasing ethanol (EtOH, Sigma Aldrich, Milan, Italy) concentration, before the analysis.

#### 3.4.4. Swelling and Stability Behaviour

To assess the swelling properties, the 3D scaffolds were immersed in PBS (10 mM) at pH levels of 5.0 and 8.0, and incubated at 37 °C. Prior to immersion, the dry weight of each scaffold (*w*_0_) was recorded. At different time points, the scaffolds were removed, gently blotted with filter paper to eliminate excess surface moisture, and weighed again to determine their swollen weight (*w_swollen_*). Measures continued until the scaffolds reached swelling equilibrium. Three swelling experiments were performed for each sample. The swelling ratio (*Q*) was calculated using the formula:

(2) $$Q=(\frac{w_{swollen}-\;w_{0}}{w_{0}})$$

Stability tests were conducted using sterile, phenol red-free Dulbecco’s Modified Eagle Medium (low glucose, DMEM; Gibco™), supplemented with antibiotics to simulate physiological conditions. The medium was prepared with or without the addition of type A collagenase (1 U/mL, Sigma Aldrich, Milan, Italy). At predetermined intervals (7, 14, and 21 days), the 3D scaffolds were retrieved from the medium, frozen at −80 °C, lyophilized, and weighed (*w_t_*). The percentage of weight loss (%) was determined using the following formula:

(3) $$Weight\;Loss\;\left(\%\right)=\left(\frac{{w_{0}-w}_{t}}{w_{0}}\right)\times \;100$$

#### 3.4.5. Dynamic Mechanical Analysis

DMA of the scaffolds was conducted using a TA-Q800 instrument (TA Instruments, New Castle, DE, USA). To mimic physiological conditions, a frequency of 1.0 Hz was applied. The tests were performed in compression mode with a 100 µm amplitude, a preload of 0.001 N, and a force track set to 125%. Measurements were carried out in a closed chamber under wet conditions at room temperature. Dehydration of the samples was considered negligible due to the short duration of each test.

#### 3.4.6. Antioxidant Properties

The ability to control ROS production was assessed by DPPH (Sigma Aldrich, Milan, Italy) test. The DPPH solution was prepared by using distilled water and 100% EtOH at a ratio of 1:1 *v/v* and a concentration of 39.5 mg/L. For 1.5 h, scaffolds (n = 6) were submerged in *DPPH* solution (3 mL) in the dark. The UV-Vis spectrophotometer was used to measure the absorbance at 517 nm. The *DPPH* radical scavenging activity (*SA_DPPH_*) was calculated using the following formula:

(4) $${SA}_{DPPH}\;\left(\%\right)=\left(\frac{{A_{blank}-A}_{sample}}{A_{blank}}\right)\times \;100$$

where *A_blank_* and *A_sample_* are the absorbance of *DPPH* solution (blank) and of the samples incubated with *DPPH* solution, respectively.

### 3.5. Cell Viability

#### 3.5.1. In Vitro Indirect Cytotoxic Assay

Mouse adipose tissue fibroblasts, L929 cells (ATCC, CCL-1 ™), were cultured in low-glucose Dulbecco’s Modified Eagle Medium (DMEM) supplemented with 10% fetal bovine serum (FBS), 1% L-glutamine, and 1% penicillin-streptomycin solution. Cells were maintained in a humidified incubator at 37 °C with 5% CO_2_.

To assess a possible indirect toxic effect of the scaffolds, L929 cells were seeded into a 96-well microtiter plate (1 × 10^4^ cells/well) and incubated for 24 h at 37 °C for attachment. Following incubation, cells were treated with 100 μL of medium previously preconditioned with UV-sterilized scaffolds. In detail, 2.5 mL of low-glucose DMEM was conditioned overnight by immersing 100 mg of each scaffold type, according to the ISO 10993–5 guidelines [72]. The conditioned media were then centrifuged at 10,000× *g* for 15 min, and the supernatants were used for cell treatments. Thus, cells were grown in scaffold-conditioned media for 24 h at 37 °C. Each condition was tested in biological triplicate. After treatment, cell viability was assessed using the 3-(4,5-dimethyl-2-thizolyl)- 2,5-diphenyl-2H-tetrazolium bromide MTT assay [73]. Briefly, 10 µL of MTT solution (0.5 mg/mL in PBS) was added to each well and incubated for 3 h at 37 °C in a humidified atmosphere. Subsequently, the supernatant was removed, and formazan crystals were dissolved by adding 100 µL of isopropanol and incubating for 1 h. Absorbance was measured at 570 nm using a microplate reader (BioTek Synergy HT Multi-Mode Microplate Reader, Agilent). Cell viability was expressed as a percentage of viable cells, calculated as the ratio between the mean absorbance of each sample and the mean absorbance of the control (untreated cells).

#### 3.5.2. In Vitro Biocompatibility Assay

Fibroblasts (L929) (previously described in Paragraph 3.5.1) were employed to investigate the effects of direct scaffold–cell interactions on cell proliferation. UV-sterilized scaffolds were placed into 96-well microtiter plates, and cells (8 × 10^4^ in 20 µL) were seeded onto the upper surface of each scaffold. The plates were incubated for 30 min at 37 °C with 5% CO_2_ to allow initial cell attachment. Subsequently, 200 µL of complete growth medium was added to fully immerse the scaffolds; plates were then incubated for an additional 24 h to allow complete cell attachment (time 0).

Cell viability was evaluated at multiple time points (time 0, 3, 7, 14, and 21 days) using the MTT assay. Scaffolds were transferred into empty wells and treated with 10 µL of MTT solution (0.5 mg/mL in PBS), allowing for scaffold penetration over a 5-min incubation. Afterward, complete culture medium was added, and the assay was performed as described in Paragraph 3.5.1. Cell viability was expressed as a percentage of viable cells, calculated as the ratio between the mean absorbance of each sample and the mean absorbance of the control (cells at time 0).

### 3.6. Antimicrobial Assay

The antimicrobial activity of the scaffolds was tested against the human pathogens *S. aureus* ATCC 6538 and the methicillin-resistant *S. aureus* (MRSA) [74]. Each tested bacterium was plated on commercial cation-adjusted Mueller-Hinton (MH) agar (Condalab, Madrid, Spain) and incubated overnight at 37 °C. Subsequently, 2–3 colonies were picked to inoculate a tube containing 3 mL of MH broth, then incubated overnight at 37 °C with shaking. After the incubation, 20 µL of the inoculum was transferred to 3 mL of fresh medium until it reached 0.5 McFarland (ca. 1–2 × 10^8^ CFU/mL). This bacterial suspension was further diluted 1:300 to prepare the final inoculum with a density of ca. 5 × 10^5^ CFU/mL. For the antimicrobial assay, 10 mg of each UV-sterilised scaffold was placed in tubes containing 2 mL of the diluted inoculum and incubated at 37 °C overnight. Tubes without scaffold were used as bacterial growth controls (CTRL); methacrylated gelatin (GelMA) [75] was used as a negative control. After incubation, a 100 µL aliquot was taken from each tube to prepare a 10-fold serial dilution (from 10^−1^ to 10^−8^) in sterile water for subsequent analysis. Subsequently, 10 µL from each dilution were spot-plated onto MH agar plates, and the plates were incubated at 37 °C overnight. After the incubation, the colonies were counted, and the CFU were compared to the control, CTRL. The antimicrobial activity of the scaffolds was quantified by calculating the log reduction according to the following formula:

(5) $$Log\;reduction=-\log_{10}\left(\frac{CFU\;Treated}{CFU\;Control}\right)$$

### 3.7. Statistical Analysis

Statistical analysis of variance of the means was assessed by *one-way* or *two-way* ANOVA and Bonferroni post hoc test (^§^ *p* < 0.1; * *p* < 0.01; ^◦^ *p* < 0.001, ^#^ *p* < 0.0001), by considering as control neat CS scaffold for each concentration, for stability, mechanical and antioxidant tests. Statistical analysis was performed using *one-way* ANOVA, followed by Dunnett’s post hoc test for multiple comparisons, for the indirect assay and antimicrobial assay. Statistical significance was assessed relative to the control group. Moreover, Student’s *t*-test was used to determine statistical differences in cell viability between day 3 and all the subsequent time points (days 7, 14, and 21). Day 3 was used as the reference point, since it showed the lowest viability, with increasing values reported at later time points.

## 4. Conclusions

Herein, the present work investigated the use of *Dicentrarchus labrax* as a source of marine collagen for wound healing applications. To this end, after successfully extracting collagen (COL), it was blended with chitosan (CS), in different proportions, for the production of scaffolds by the freeze-drying technique.

ATR-FTIR confirmed that higher COL loading in CS scaffolds lead to a reinforced network characterized by intermolecular hydrogen bonding. This result was also confirmed by DSC. In particular, the consistently lower T_d_ of CS4 compared to CS2 likely may be ascribed to a heterogeneity in network formation. Indeed, higher viscosity in CS4 may slow freezing and restrict TPP diffusion, leading to regions with more loosely bound water that dehydrate at lower temperatures. In contrast, CS2 formed a more homogeneous, efficiently crosslinked structure that binds water more strongly. The presence of COL, at a higher concentration, also improved thermal stability.

The morphological analysis revealed scaffolds with an open-pore structure with high interconnectivity, essential for cell migration and proliferation. The addition of collagen enhanced the porosity and pore size of CS scaffolds, which is crucial for tissue growth, oxygen transport, and waste removal.

Swelling behavior was evaluated at pH 5.0 and 8.0, simulating the extreme pH conditions of the overall wound healing process. Results demonstrated that CS with the highest concentration (CS4) scaffolds showed varying swelling ratios depending on the pH, with COL increasing swelling at lower pH values. The scaffolds exhibited good stability, maintaining their 3D structure without collapsing over time. The degradation rate was assessed using collagenase, with CS/COL scaffolds showing weight loss proportional to the collagen concentration, suggesting that collagen provided additional structural integrity and protection. Mechanical properties were also assessed using dynamic mechanical analysis (DMA), revealing a significant influence of both CS and COL concentrations on the storage modulus. Higher concentrations of both materials resulted in scaffolds with suitable mechanical strength to withstand forces during wound healing. It is worth noting that the apparent discrepancy between thermal and mechanical results can be explained. T_d_ reflects water–polymer interactions, whereas mechanical properties depend mainly on polymer content and scaffold morphology. Thus, although CS2 bound water more strongly (higher T_d_), and appeared more efficiently crosslinked, CS4 achieved the highest mechanical properties and stability, due to its higher CS concentration, thicker pore walls, and denser microstructure, as confirmed by SEM. Overall, T_d_ data agree with ATR-FTIR, SEM, swelling and mechanical results and support a model in which COL acts first as a local plasticizer at low loading and as a network-forming, water-retaining agent at higher loading.

Finally, antioxidant activity, as measured by the DPPH assay, was also assessed. It indicated that the scaffolds possess the ability to scavenge free radicals, an important feature for wound healing.

Biocompatibility was evaluated using murine fibroblasts (L929 cells), and the results showed that the scaffolds did not release any cytotoxic components and supported cell attachment and growth. The inclusion of marine COL enhanced fibroblast proliferation, particularly in the CS4/COL1.0 scaffold, which reached 87% cell viability on day 21. SEM images confirmed good cell attachment and spreading on the scaffold surfaces.

Finally, antimicrobial activity was assessed against *S. aureus* and methicillin-resistant *S. aureus* (MRSA). All scaffolds exhibited significant bactericidal activity, indicating that the presence of marine collagen in the scaffolds maintains the already known antimicrobial activity of chitosan.

The overall results indicate that scaffold properties can be finely tuned by adjusting the relative amounts of CS and COL. For acute wounds, where rapid resorption, high porosity, and fast healing are required, CS2/COL0.5 is most suitable, offering excellent porosity, high swelling capacity, and enhanced cell adhesion with moderated degradation. For chronic wounds, which demand prolonged mechanical support and gradual degradation, CS4/COL1.0 provides the best compromise, combining superior mechanical strength and structural stability with the bioactive contribution of collagen.

In conclusion, CS and CS/COL scaffolds represent promising biocompatible and antimicrobial materials for skin tissue engineering, with tunable properties that make them well-suited for different wound healing applications.

## Figures and Tables

**Table 1:** Characteristic Attenuated total reflection Fourier-transform infrared (ATR-FTIR) bands.

Wavenumber (cm^−1^)	Main Assignment	Contribution from CS	Contribution from COL
~3300	O–H and N–H stretching (H-bonding)	–OH and protonated –NH_2_ groups	Amides (–NH)
~2932	Aliphatic C–H stretching	CS backbone	Minimal
~1631–1642 (Amide I)	C=O stretching (peptide bond)	Weak (residual acetyl groups)	Strong (peptide bonds)
~1547 (Amide II)	N–H bending + C–N stretching	Weak	Strong
~1236–1237 (Amide III)	C–N stretching + N–H bending	Weak	Strong
~1150	Asymmetric C–O–C bridge	Strong	–
1070–1030	C–O stretching (alcohol and C–O–C groups)	Strong	–

**Table 2:** Nomenclature of the scaffolds produced by freeze-drying technique.

Sample	Chitosan (CS) (mg/mL)	Collagen (COL) (mg/mL)
CS2	20	-
CS2/COL0.5		5
CS2/COL1.0		10
CS4	40	-
CS4/COL0.5		5
CS4/COL1.0		10

## Data Availability

The authors declare that all data supporting the findings of this study are available within the paper; source data for the figures in this study are available from the authors upon request.
